# Survival Prediction in Intrahepatic Cholangiocarcinoma: A Proof of Concept Study Using Artificial Intelligence for Risk Assessment

**DOI:** 10.3390/jcm10102071

**Published:** 2021-05-12

**Authors:** Lukas Müller, Aline Mähringer-Kunz, Simon Johannes Gairing, Friedrich Foerster, Arndt Weinmann, Fabian Bartsch, Lisa-Katharina Heuft, Janine Baumgart, Christoph Düber, Felix Hahn, Roman Kloeckner

**Affiliations:** 1 Department of Diagnostic and Interventional Radiology, University Medical Center of the Johannes Gutenberg University Mainz, 55131 Mainz, Germany; lukas.mueller@unimedizin-mainz.de (L.M.); aline.maehringer-kunz@unimedizin-mainz.de (A.M.-K.); christoph.dueber@unimedizin-mainz.de (C.D.); felix.hahn@unimedizin-mainz.de (F.H.); 2 Department of Internal Medicine, University Medical Center of the Johannes Gutenberg University Mainz, 55131 Mainz, Germany; simonjohannes.gairing@unimedizin-mainz.de (S.J.G.); friedrich.foerster@unimedizin-mainz.de (F.F.); arndt.weinmann@unimedizin-mainz.de (A.W.); 3 Department of General, Visceral and Transplant Surgery, University Medical Center of the Johannes Gutenberg University Mainz, 55131 Mainz, Germany; fabian.bartsch@unimedizin-mainz.de (F.B.); lisa-katharina.heuft@unimedizin-mainz.de (L.-K.H.); janine.baumgart@unimedizin-mainz.de (J.B.)

**Keywords:** intrahepatic cholangiocarcinoma, survival prediction, risk scoring, machine learning, artificial intelligence, artificial neural network, Fudan score

## Abstract

Several scoring systems have been devised to objectively predict survival for patients with intrahepatic cholangiocellular carcinoma (ICC) and support treatment stratification, but they have failed external validation. The aim of the present study was to improve prognostication using an artificial intelligence-based approach. We retrospectively identified 417 patients with ICC who were referred to our tertiary care center between 1997 and 2018. Of these, 293 met the inclusion criteria. Established risk factors served as input nodes for an artificial neural network (ANN). We compared the performance of the trained model to the most widely used conventional scoring system, the Fudan score. Predicting 1-year survival, the ANN reached an area under the ROC curve (AUC) of 0.89 for the training set and 0.80 for the validation set. The AUC of the Fudan score was significantly lower in the validation set (0.77, *p* < 0.001). In the training set, the Fudan score yielded a lower AUC (0.74) without reaching significance (*p* = 0.24). Thus, ANNs incorporating a multitude of known risk factors can outperform conventional risk scores, which typically consist of a limited number of parameters. In the future, such artificial intelligence-based approaches have the potential to improve treatment stratification when models trained on large multicenter data are openly available.

## 1. Introduction

Intrahepatic cholangiocarcinoma (ICC) is the second most common type of primary liver cancer after hepatocellular carcinoma (HCC). The incidence of ICC is low in Western countries but has been rising continuously in recent decades [1,2,3,4]. Unfortunately, symptoms of ICC mostly appear in the late stages of the disease. Thus, resection, which is the only curative treatment option, is not possible in the majority of cases [5]. In addition, recurrence rates after initial resection exceed 60% [6]. Novel treatment options have become available in recent decades, and knowledge on prognostic factors is growing [7,8]. This is allowing treatment during the course of disease to be more individualized. Due to this growing heterogeneity, risk prediction is becoming more and more difficult.

Conventional scoring models for risk stratification have been proposed by several groups [9,10,11]. Most of them were designed primarily for patients undergoing curative resection and use histopathological factors, such as microvascular invasion or tumor grading, which are only available postoperatively [9,10,11]. Even though all attempts have initially shown promising results, they have failed external validation and have not entered clinical use [12,13]. The only available score for all patients regardless of subsequent treatment is the Fudan score [14]. The tumor itself plays a major role in this score, which comprises tumor diameter, number of lesions, tumor boundary, level of tumor marker carbohydrate antigen 19-9 (CA19-9), and serum alkaline phosphatase (AP) level. All of these parameters are easily assessable during the initial patient work-up. Thus, the score provides an ab initio method for assisting clinicians in patient stratification. However, the score has never been externally evaluated for patients with ICC regardless of the initial therapy.

All of the conventional scoring approaches are easy to calculate and may be comprehensible, but it remains questionable whether such a limited number of parameters is sufficient to achieve reliable prediction for clinical decision making.

An alternative to conventional scoring systems is the increasing integration of machine learning (ML) approaches into risk assessment. Systems based on ML have proven their feasibility and superiority compared to conventional scoring systems in survival prediction for hepatocellular and colorectal cancer [15,16,17]. Thus far, for ICC, a few similar approaches have been tried for the subgroup of resected patients in order to calculate the risk of recurrence, to decide upon adjuvant treatment, and to predict the median overall survival (OS) [18,19,20]. For these decisions, such approaches outperformed the conventional scoring systems.

We hypothesize that the main reason for the superiority of ML algorithms over conventional approaches is based on the possibility of including a wider range of parameters. In particular, artificial neural networks (ANNs) are ideal to include a wide range of different parameters and offer flexible scalability when complexity increases [15].

Thus, this study attempted to build an ANN based on a much broader range of parameters in order to improve prediction for patients with ICC prior to making decisions on treatment. In a second step, we evaluated our newly designed model against the conventional Fudan score in a head-to-head comparison.

## 2. Materials and Methods

The study was approved by the responsible ethics committee (permit number 2018—13618, date of approval: 15 October 2018). Patient records and clinical information were de-identified before analysis. Additional examinations were not performed. The TRIPOD and STROBE guidelines were followed for the construction of the manuscript (Supplementary Tables S1 and S2) [21,22].

### 2.1. Patients

**Figure 1:**
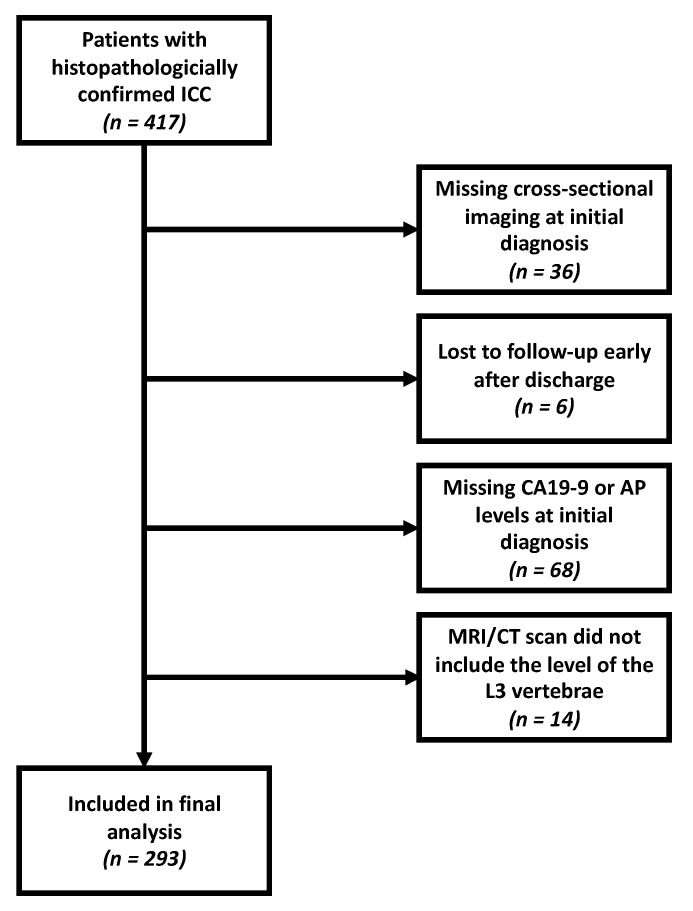
Flow diagram showing the reasons for exclusion from the study. CA19-9, carbohydrate antigen 19-9. AP, alkaline phosphatase. MRI, magnetic resonance imaging. CT, computed tomography.

Between January 1997 and January 2018, 417 patients with histopathologically confirmed ICC were referred to our tertiary care center. After retrospectively identifying these patients using established clinical registry software, 124 were excluded for the reasons described in Figure 1. The final analysis was performed on the remaining 293 patients.

### 2.2. Diagnosis, Treatment and Follow-Up

Histopathological diagnosis was performed based on the European Association for the Study of the Liver guidelines for the diagnosis and management of ICC [7]. All patients underwent contrast-enhanced computed tomography (CT) or magnetic resonance imaging (MRI) for treatment planning and staging. Prior to making a treatment decision, all patients underwent an extensive discussion with an interdisciplinary tumor board consisting of visceral surgeons, hepatologists/oncologists, diagnostic and interventional radiologists, pathologists, and, if needed, radiation therapists. Follow-up comprised clinical examination, blood sampling, and cross-sectional imaging.

### 2.3. Data Acquisition

Patient data were acquired using the clinical registry unit (CRU). The CRU is an established registry that prospectively collects all patients with liver cancer treated at our tertiary care referral center [23]. The data for this study were retrospectively collected and analyzed. The CRU dataset includes all baseline characteristics, including demographic data, serological parameters, treatment-related parameters, and information on the tumor burden, including size and number of intrahepatic lesions, tumor boundary type, translobar and extrahepatic spread, and the presence of nodal and distant metastases. Standardized cut-offs for the serological and imaging parameters were derived from the original Fudan score [14]. In particular, the tumor boundary was assessed as described in the original paper [14]. Translobar spread was specified as tumor expansion per continuitatem or as intrahepatic metastasis in more than one lobe. According to the current AJCC/UICC TNM staging system, an extrahepatic spread exists if the tumor perforates the viscera of the liver and/or infiltrates adjacent organs [24]. The psoas muscle index (PMI) was defined as the total area of the psoas muscle at the level of the L3 vertebra divided by the squared body height [25,26]. For the definition of high and low PMI, we used cut-offs derived previously by our group using optimal stratification. In the resected group, “low” was defined as ≤5.7 cm^2^/m^2^ for men and ≤5.1 cm^2^/m^2^ for women, whereas in the non-resected subgroup, the values were ≤5.5 cm^2^/m^2^ for men and ≤4.8 cm^2^/m^2^ for women [25]. In the case of missing data, the information was updated using the radiology information system and the laboratory database. The primary endpoints were median OS and the 1-year survival rate. OS was defined as the time interval between the initial diagnosis and death or last follow-up. Death dates were acquired and updated with information from the appropriate Residents’ Registration Offices.

### 2.4. Calculation of the Fudan Score

**Figure 2:**
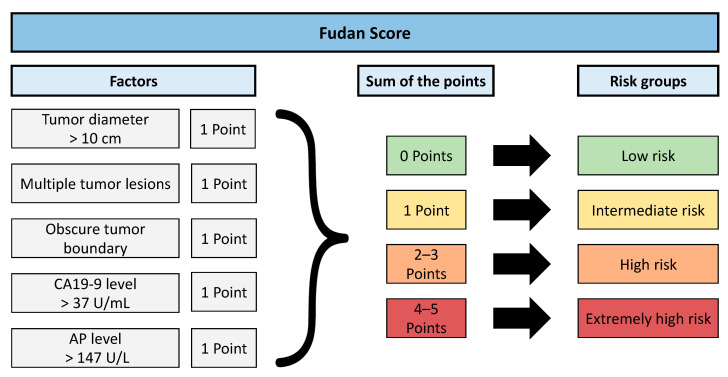
Calculation of the Fudan score. CA19-9, carbohydrate antigen 19-9. AP, alkaline phosphatase.

The Fudan score was calculated as described in its original publication [14]. Figure 2 summarizes the included parameters, their weights, and the grouping used for risk stratification.

### 2.5. Design of the Neural Network

The neural network was built using Tensorflow (https://www.tensorflow.org/, version 1.13.0, Google LLC, Mountain View, USA, accessed on 31 January 2021) and Keras (https://keras.io/, version 2.2.0, Francois Chollet, Google LLC, Mountain View, CA, USA, accessed on 31 January 2021). It consisted of three fully connected hidden layers with 16, 12, and 8 nodes, respectively. To simplify, each of the hidden layers is a specific, complex mathematical function with different functional characteristics and designed to produce a defined output. By the conjunction of each defined output from each layer, a neural network can make a specific, overall prediction [27]. Rectified linear unit (ReLU) was used as the activation function on all hidden layers and sigmoid classification for the final output layer. To prevent overfitting, we used L2-regularization. Standardization was performed on all input parameters by subtraction of the mean and division by the standard deviation.

**Figure 3:**
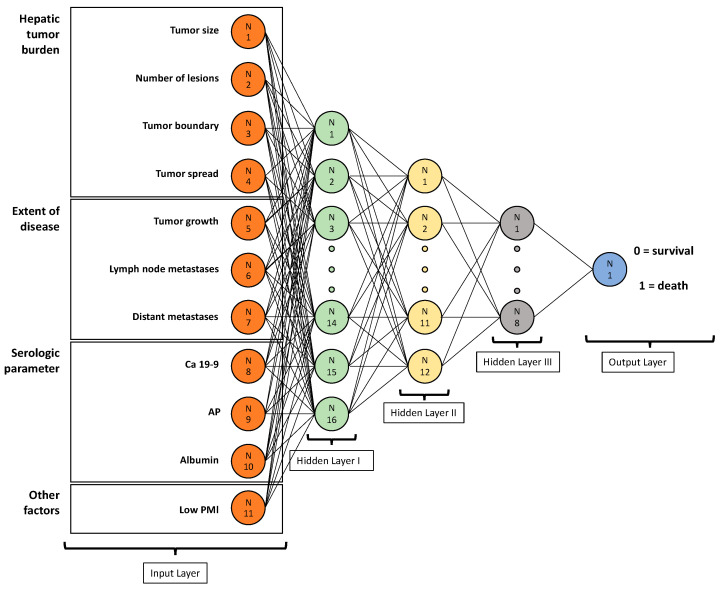
Visualization of the created artificial neural network.

As input nodes, we included all factors of the Fudan score (tumor diameter, number of lesions, tumor boundary, CA19-9 and AP serum levels) as well as potentially meaningful parameters (tumor spread, extrahepatic tumor extension, the presence of lymph node and distant metastases). Furthermore, we included a low PMI as a parameter representing the patient’s overall condition and the albumin level as a parameter representing the hepatic reserve. The final output results for the network were survival and death one year after initial diagnosis. The ANN is visualized in Figure 3.

### 2.6. Training and Validation of the ANN

**Figure 4:**
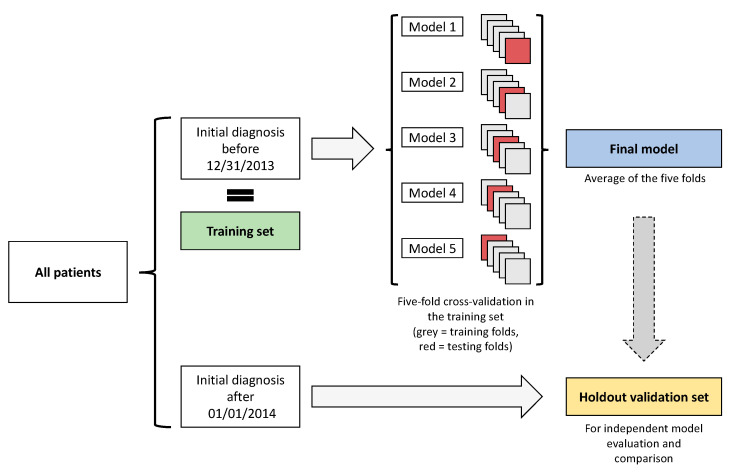
Visualization of the created artificial neural network.

For an 80:20 split, all patients with an initial diagnosis before 31 December 2013 (*n* = 233, 80%) were allocated to the training set. Patients with an initial diagnosis afterwards (*n* = 60, 20%) formed the holdout validation set. As suggested elsewhere, the holdout validation dataset was only used for final evaluation of the models and their comparison [15]. In the training set, a five-fold cross-validation approach was used to maximize the training capabilities of the ANN. Figure 4 provides an overview on the process used for model training and validation.

### 2.7. Statistical Analysis

Statistical analyses and graphic design were performed in R 4.0.3 (A Language and Environment for Statistical Computing, http://www.R-project.org, R Foundation for Statistical Computing, Vienna, Austria, accessed on 31 January 2021). Continuous data were reported as medians and ranges. Categorical and binary baseline parameters were reported as absolute numbers and percentages. Fisher’s exact tests, chi-squared tests, or Mann–Whitney *U* tests were used for *p*-value computations between the training and test sets, where appropriate. Survival analysis was performed using the packages “survminer” (https://cran.r-project.org/package=survminer, accessed on 31 January 2021, R Foundation for Statistical Computing, Vienna, Austria) and “survival” (https://CRAN.R-project.org/package=survival, accessed on 31 January 2021, R Foundation for Statistical Computing, Vienna, Austria). Strata were compared by log-rank testing. Univariate and multivariate Cox proportional hazard regression models assessing hazard ratios (HRs) and corresponding 95% confidence intervals (CIs) were performed to determine the influence of risk factors on the median OS. Performance of the Fudan score in individual survival prediction was assessed using Harrell’s concordance index (C-Index) [28]. A C-Index of 0.5 indicates no predictive ability and 1.0 indicates perfect predictive power. The performance of the Fudan score and the ANN model for predicting the 1-year survival rate was measured using the area under the receiver operating characteristic curve (AUC). The AUC ranges from 0 to 1: 0.5 indicates no predictive ability, 1.0 indicates perfect prediction, and <0.5 indicates “anti-prediction”. A *p*-value of <0.05 was considered significant.

## 3. Results

### 3.1. Baseline Characteristics

Of the 293 patients analyzed in this study, 176 (60.1%) were males and 117 (39.9%) were females. The median age at the initial TACE treatment was 66 years. Median follow-up for all patients was 12.6 months. Both the training and the validation set had no statistical differences in their baseline characteristics. Median OS of the patients in the training set was 13.1 months (95% CI 10.1–16.7 months) and 16.3 months for patients in the validation set (95% CI 11.1–22.8 months). Table 1 displays the baseline characteristics of the cohort.

### 3.2. Risk Factor Identification for the ANN-Based Model

To identify possible risk factors for inclusion in the ANN model, univariate Cox hazard regression was performed. Except for age > 60 years, a parameter which is included in the MEGNA score [11], all investigated risk factors reached highly significant *p*-values (Table 2). Therefore, all of these factors were used in the input layer of the ANN model.

### 3.3. Predictive Performance of the ANN

**Figure 5:**
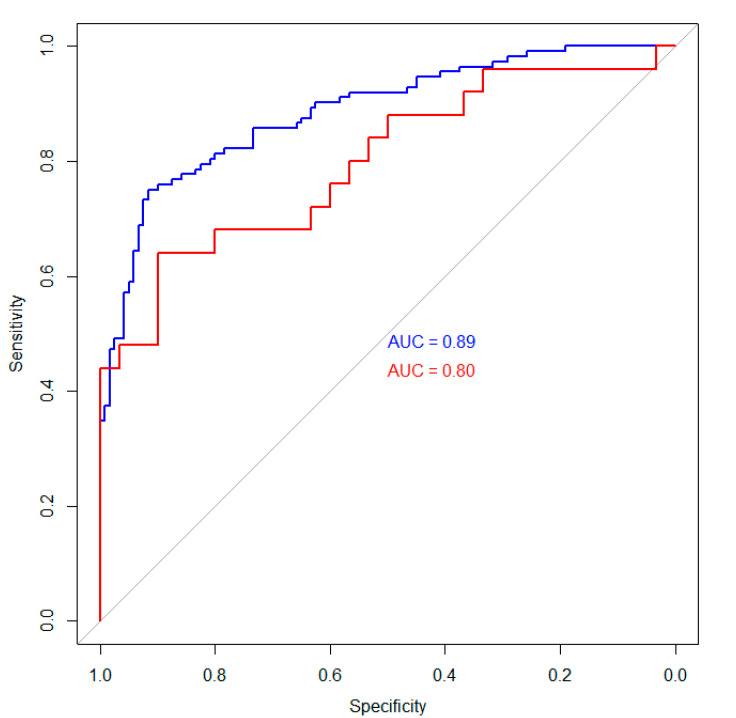
Visualization of the created artificial neural network. Receiver operating characteristic curves for the training (blue) and validation (red) sets.

For the ANN, the AUC was 0.89 (95% CI 0.84–0.93) for the training set and 0.80 (95% CI 0.68–0.92) for the holdout validation set (Figure 5).

### 3.4. Predictive Performance of the Fudan Score

**Figure 6:**
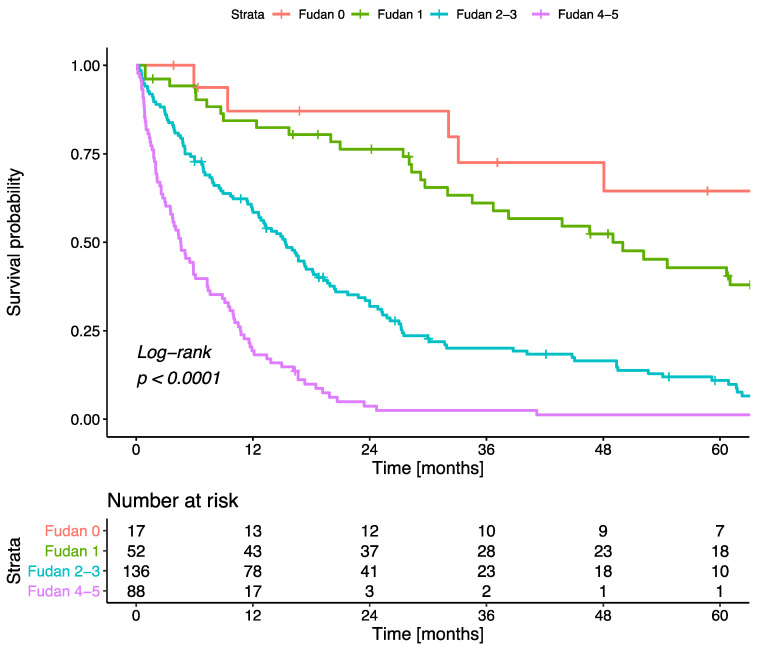
Kaplan–Meier curves of overall survival stratified according to Fudan score.

In a second step, we performed a head-to-head comparison of our newly developed ANN and the conventional Fudan score. Of the 293 patients, 17 (5.8%) had a low, 52 (17.8%) an intermediate, 136 (46.4%) a high, and 88 (30.0%) an extremely high Fudan score. The median OS was 69 months, 50 months, 15 months, and 5 months in the low-, intermediate-, high-, and extremely high risk groups, respectively (log-rank *p*-value < 0.001, Figure 6).

**Figure 7:**
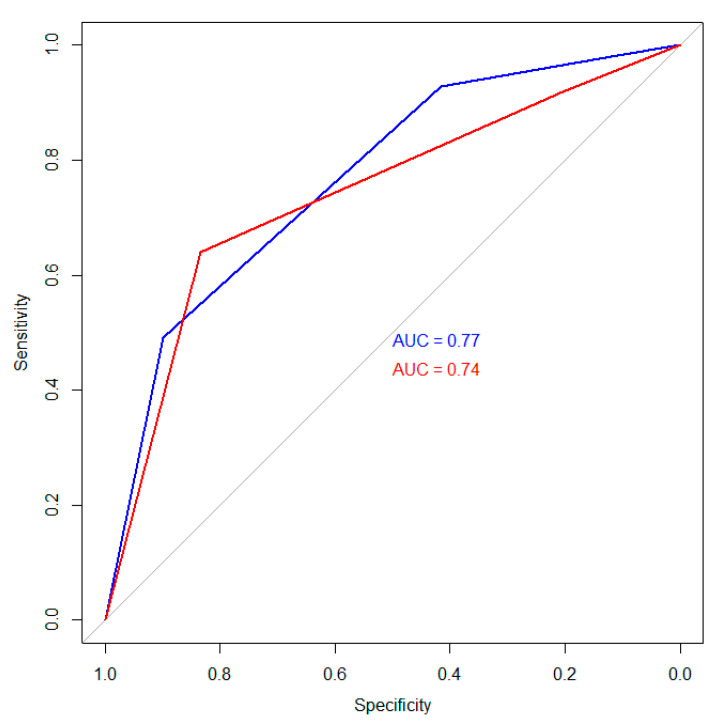
Receiver operating characteristic curves for the training (blue) and validation (red) sets using the Fudan score.

Regarding individual risk prediction, the Fudan score yielded a Harrell’s C-Index of 0.69 and an AUC for predicting 1-year survival probability of 0.77 (95% CI 0.71–0.82) for the training set and 0.74 (95% CI 0.61–0.87) for the holdout validation set (Figure 7).

Comparing both models, the AUC differed significantly for the training cohort (0.89 vs. 0.77, *p* < 0.001), but the difference between both AUCs for the validation set did not reach significance (0.80 vs. 0.74, *p* = 0.24).

## 4. Discussion

In this study, we evaluated the feasibility of an ANN for ab initio risk prediction in patients with ICC. In a second step, we evaluated the Fudan score and performed a head-to-head comparison. In summary, the ANN reached an AUC of 0.89 in the training set and therefore outperformed the Fudan score (0.77) significantly (*p* < 0.001). In the validation set, the ANN was also superior compared to the Fudan score (0.80 vs. 0.74). However, this difference did not reach significance (*p* = 0.24), which might be attributable to the smaller sample size of the validation set. However, ANN models have excellent scalability; therefore, novel risk factors can easily be added to the developed model. Hence, these approaches will further improve risk prediction in patients with ICC.

Thus far, several scoring systems have been developed, especially for patients who have undergone tumor resection. The Hyder nomogram depends on tumor size, nodal status, vascular invasion, multifocality, presence/absence of cirrhosis, and age [9]. The Wang nomogram includes carcinoembryonic antigen and CA19-9 levels, vascular invasion, nodal status, and direct invasion or local metastasis, as well as tumor size [10]. The MEGNA score stratifies risk groups using the parameters multifocality, extrahepatic tumor extension, tumor grading, lymph node metastasis, and age [11]. Despite promising initial results, they all failed in external validation; though the Hyder nomogram had a C-Index of 0.69 in the derivation cohort, in an external validation by Doussot et al., the C-Index only reached 0.63. In the same study, the Wang nomogram reached superior values in estimating prognosis (C-Index 0.72). In two recent evaluations, the MEGNA score was found to be a useful stratification tool but failed in individual risk prediction [13,29]. Thus, none of the scores were implemented in the daily clinical routine.

The only scoring system available for patients regardless of histopathological factors is the Fudan score. This score consists of five common parameters assessed during standard work-up at the time of initial diagnosis and is not based on histopathological factors [14]. In a previous study by our group, all the included factors correlated with an impaired survival in our patient cohort [25]. Thus, the high discriminative ability (*p* < 0.001) of the score in this study is not surprising. However, regarding individual survival prediction, the corresponding C-Index was only moderate (0.69), and 1-year survival prediction reached values of 0.77 for the training set and 0.74 for the validation set, which can be classified as a “fair prediction” [30,31]. One reason for the only moderate predictive ability of the Fudan score in our patient cohort might be the fact that we calculated the score regardless of the initial treatment. In the original publication, the authors developed the score on a population of resected cases and evaluated its performance on a small set of unresected patients.

All of the above-mentioned stratification systems rely on well-known clinical, histopathological, serological, and imaging-derived factors. However, they may not cover the clinical complexity because they are all based only on a few, mainly tumor burden-associated factors. Knowledge about novel risk factors, such as the tumor microenvironment, the influence of inflammation and immune reactions, body composition assessment, tumor standardized uptake in hybrid positron emission tomography/computed tomography imaging, and image-based texture analysis has continuously been increasing [25,32,33,34,35,36,37]. Therefore, the integration of these factors into scoring systems has great potential. For a successful translation into daily patient care, ML-based approaches offer a solution for the conjunction of well-known risk factors and this emerging knowledge. In addition, automated parameter processing using ML-based approaches becomes more applicable due to the continuous growth of digitization in the clinical infrastructure and electronic availability of patient data. In the future, dedicated software pipelines based on these approaches will enable automatic risk prediction.

However, ML-based studies on survival prediction in patients with ICC are scarce. Thus far, three attempts have been made: Focusing on tumor burden and the relationship between tumor size and number, Bagante et al. used a classification and regression tree model (CART) to identify prognostic groups of patients after curative-intent resection [18]. With their CART model, the group was able to visualize the hierarchical association between tumor burden and other clinical and histopathological factors. Li et al. applied different decision tree- and random forest-based ML algorithms to identify the most important risk factors for patients with ICC after resection [19]. In a second step, they created a novel scoring system based on the T and N categories of the ICC staging framework in the AJCC 8th edition, namely, carcinoembryonic antigen, CA19-9, alpha-fetoprotein, and prealbumin. Although their so-called EHBH-ICC score outperformed the AJCC 8th and LCSGJ staging systems, the final model’s C-Index was only moderate (0.69 for training and 0.67 for internal validation). The latest attempt by Jeong et al. achieved better values: in contrast to the two attempts before, but similar to our study, they used a Tensorflow deep learning algorithm to create a scoring system based on the wide range of four postoperative histopathological, six serological, and two etiological factors [20]. This system yielded an AUC of 0.78 in the original study and was more accurate than the AJCC staging system (0.60). In combination with our results, this supports our hypothesis that the inclusion of more risk factors enhances individual survival prediction.

Compared to other ML approaches and conventional scoring systems, the main advantage of ANNs may be that a multitude of different variables can be included quickly and the networks are easily scalable when novel parameters are integrated and complexity increases [15]. ANNs have the disadvantage of being kind of “black boxes” with complex interactions between included parameters and subsequent layers [15]. Furthermore, ANNs cannot deal with missing values. Thus, datasets have to be as complete as possible. In the future, this bias may be attenuated, as the digitization of medical records is continuously progressing and more and more parameters are automatically assessed. However, our results should only be interpreted as a proof of feasibility due to the single-center design and missing external validation. Hence, large-scale validation studies are mandatory in the future.

One point that further stresses the potential of artificial intelligence-based approaches for survival prediction is the following fact: even though there was considerable heterogeneity regarding initial treatment, our approach reached a strong prognostic ability—even when applied at the very beginning of the patient’s clinical history.

Our study has several limitations: First, the dataset was acquired in a retrospective manner and the final sample size was only moderate (*n* = 293) due to the monocentric nature of the study. However, the number of included patients was comparable to other studies examining the role of risk prediction and stratification for patients with ICC [9,10,11,12,14]. Second, as incidence is low in Western countries, the recruitment period was relatively long. In the meantime, significant improvements have been made in treatment, especially for patients with an unresectable tumor burden, and indication criteria have changed tremendously [8,38]. To reduce this bias, we actively decided to choose patients with an initial diagnosis in 2014 or later for the validation set. Third, we included only patients with complete datasets and actively decided against imputing missing values. Thus, we were not able to include important prognostic factors such as the Eastern Cooperative Oncology Group Performance Status or inflammation parameters such as the neutrophil to lymphocyte ratio or the platelet to lymphocyte ratio as the determination of these factors has not been a standard for patients treated before 2010. Therefore, the integration of these parameters would have considerably reduced the number of patients included into final analysis. However, especially the growing knowledge on inflammation indices offers great potential for survival prediction in patients with intrahepatic cholangiocarcinoma as they are easily available pre-operative serum markers. Fourth, for the sake of a clear methodology, we decided to use an 80:20 split based on the time of the initial diagnosis. However, as mentioned above, significant improvements have been made in treatment and in indication criteria. Therefore, the allocation according to the initial diagnosis date could have introduced a bias. However, even though treatment options evolved during the study period, our approach outperformed the Fudan score clearly for the validation set and reached a good predictive ability. Fifth, scoring systems derived from a single-center cohort of patients face the problem of “overfitting”. “Overfitting” describes “a phenomenon occurring when a model maximizes its performance on some set of data, but its predictive performance is not confirmed elsewhere due to random fluctuations of patients’ characteristics in different clinical and demographical backgrounds” [39]. Multicenter studies and the inclusion of patients with different ethnic backgrounds will attenuate this bias. Such studies would also enable us to approach the full capability of an ANN-based model.

## 5. Conclusions

ML-based approaches and especially ANNs offer the possibility of integrating a broad range of different patient parameters into risk prediction. This study proved the feasibility of this approach for patients with ICC prior to treatment. The ANN outperformed conventional risk scoring, leading to the conclusion that especially the inclusion of more risk factors offers a great potential for survival prediction. To reach the full capability of such approaches, large multicenter clinical databases are needed. Afterwards, such “big data”-based ANNs could easily be implemented into, for example, web-based risk calculations and integrated into the clinical routine workflow in order to support clinicians in daily decision making.

## Figures and Tables

**Table 1:** Baseline characteristics of the patient cohort.

		All (*n* = 293)	Training Set (*n* = 233)	Validation Set (*n* = 60)	*p*-Value
Age, years	Median (IQR)	66.0 (57–73)	66.1 (57–73)	65.4 (57–73)	0.79 ^†^
Sex, *n* (%)	Male	176 (60.1)	143 (61.4)	33 (55.0)	0.38 ^‡^
	Female	117 (39.9)	90 (38.6)	27 (45.0)	
Number of intrahepatic lesions, *n* (%)	1	174 (59.4)	135 (57.9)	39 (65.0)	0.07 ^††^
	2	30 (10.2)	28 (12.0)	2 (3.3)	
	3	14 (4.8)	14 (6.0)	0 (0.0)	
	4	14 (4.8)	10 (4.3)	4 (6.7)	
	≥5	61 (20.8)	46 (19.8)	15 (25.0)	
Tumor size, mm	Median (IQR)	89 (56–146)	88 (56–145)	98 (55–153)	0.90 ^†^
Tumor boundary type, *n* (%)	Distinct	105 (35.8)	88 (37.8)	17 (28.3)	0.23 ^‡^
	Obscure	188 (64.2)	145 (62.2)	43 (71.7)	
Tumor spread, *n* (%)	Unifocal or intra-lobar metastasis	206 (70.3)	161 (69.1)	45 (75.0)	0.43 ^‡^
	Translobar metastasis	87 (29.7)	72 (30.1)	15 (25.0)	
UICC T stage ≥ 3, *n* (%)	Yes	64 (21.8)	51 (21.9)	13 (21.7)	0.58 ^‡^
	No	229 (78.2)	182 (78.1)	47 (78.3)	
Lymph node metastases, *n* (%)	Yes	88 (30.0)	70 (30.0)	18 (30.0)	1.00 ^‡^
	No	205 (70.0)	163 (70.0)	42 (70.0)	
Distant metastases, *n* (%)	Yes	74 (25.3)	57 (24.5)	17 (28.3)	0.62 ^‡^
	No	219 (74.7)	176 (75.5)	43 (71.7)	
AP serum levels, U/L	Median (IQR)	161 (102–290)	158 (99–306)	168 (116–256)	0.50 ^†^
Ca 19-9 serum levels, U/mL	Median (IQR)	80 (22–800)	82 (18–773)	70 (31–1046)	0.46 ^†^
Albumin, g/dL	Median (IQR)	3.8 (3.4–4.2)	3.9 (3.4–4.2)	3.8 (3.4–4.1)	0.29 ^†^
Initial therapy	Resection	143 (48.8)	116 (49.8)	27 (45.0)	0.19 ^††^
	Ablation	3 (1.0)	1 (0.4)	2 (3.3)	
	TACE *	14 (4.8)	9 (3.9)	5 (8.3)	
	SIRT *	29 (9.9)	24 (10.3)	5 (8.3)	
	Chemotherapy only	54 (18.4)	41 (17.6)	13 (21.7)	
	BSC	50 (17.1)	42 (18.0)	8 (13.3)	

* Of the 43 patients who received transarterial treatments, 20 received additional chemotherapy (*n* = 12 in the training set, *n* = 8 in the validation set). UICC, union internationale contre le cancer. CA19-9, carbohydrate antigen 19-9. AP, alkaline phosphatase. ^†^ Mann–Whitney *U* test used. ^‡^ Fisher test used. ^††^ Chi-squared test used.

**Table 2:** Univariate Cox hazard regression model results.

Factor	Univariate	
	HR (95% CI)	*p*-Value
Age > 60 years	1.2 (0.9–1.6)	0.140
Max. tumor size > 10 cm	1.9 (1.5–2.5)	<0.001
Multifocality	2.0 (1.6–2.6)	<0.001
Obscure tumor boundary	2.4 (1.8–3.2)	<0.001
Translobar spread	2.9 (2.2–3.8)	<0.001
Extrahepatic tumor growth	1.6 (1.2–2.2)	<0.001
Lymph node metastases	2.1 (1.6–2.7)	<0.001
Distant metastases	4.2 (3.1–5.7)	<0.001
Ca 19-9 > 37 U/mL	2.2 (1.7–2.9)	<0.001
AP > 147 U/L	2.0 (1.5–2.5)	<0.001
Albumin < 3.5 g/dL	2.6 (2.0–3.5)	<0.001
Low PMI	1.6 (1.2–2.0)	<0.001

HR, hazard ratio. CI, confidence interval. CA19-9, carbohydrate antigen 19-9. AP, alkaline phosphatase. PMI, psoas muscle index.

## Data Availability

Data cannot be shared publicly because of institutional and national data policy restrictions imposed by the Ethics Committee of the Medical Association of Rhineland Palatinate, Mainz, Germany, since the data contain potentially identifying patient information. Data are available upon request for researchers who meet the criteria for access to confidential data.

## Supplementary Materials

The following are available online at https://www.mdpi.com/article/10.3390/jcm10102071/s1, Table S1: TRIPOD Checklist: Prediction Model Development and Validation, Table S2: STROBE Statement—Checklist of items that should be included in reports of cohort studies.
