# Supplementary material for: Survival Prediction in Intrahepatic Cholangiocarcinoma: A Proof of Concept Study Using Artificial Intelligence for Risk Assessment
Source: J Clin Med. 2021 May 12;10(10):2071. doi: 10.3390/jcm10102071 (PMC8150393; doi:10.3390/jcm10102071)
Supplement: Supplementary file 1 [file jcm-10-02071-s001.zip › jcm-1176579-supplementary.pdf]

Supplementary Materials

Table S1. TRIPOD Checklist: Prediction Model Development and Validation [1].

Section/Topic	Item		Checklist Item	Page
			**Title and abstract**	
Title	1	D;V	Identify the study as developing and/or validating a multivariable prediction model, the target population, and the outcome to be predicted.	1
Abstract	2	D;V	Provide a summary of objectives, study design, setting, participants, sample size, predictors, outcome, statistical analysis, results, and conclusions.	1
			**Introduction**	
Background and objectives	3a	D;V	Explain the medical context (including whether diagnostic or prognostic) and rationale for developing or validating the multivariable prediction model, including references to existing models.	1–2
	3b	D;V	Specify the objectives, including whether the study describes the development or validation of the model or both.	1–2
			**Methods**	
Source of data	4a	D;V	Describe the study design or source of data (e.g., randomized trial, cohort, or registry data), separately for the development and validation data sets, if applicable.	2
	4b	D;V	Specify the key study dates, including start of accrual; end of accrual; and, if applicable, end of follow-up.	3
Participants	5a	D;V	Specify key elements of the study setting (e.g., primary care, secondary care, general population) including number and location of centres.	2
	5b	D;V	Describe eligibility criteria for participants.	2
	5c	D;V	Give details of treatments received, if relevant.	6
Outcome	6a	D;V	Clearly define the outcome that is predicted by the prediction model, including how and when assessed.	4
	6b	D;V	Report any actions to blind assessment of the outcome to be predicted.	4
Predictors	7a	D;V	Clearly define all predictors used in developing or validating the multivariable prediction model, including how and when they were measured.	3–4
	7b	D;V	Report any actions to blind assessment of predictors for the outcome and other predictors.	4
Sample size	8	D;V	Explain how the study size was arrived at.	2
Missing data	9	D;V	Describe how missing data were handled (e.g., complete-case analysis, single imputation, multiple imputation) with details of any imputation method.	6
Statistical analysis methods	10a	D	Describe how predictors were handled in the analyses.	6
	10b	D	Specify type of model, all model-building procedures (including any predictor selection), and method for internal validation.	4-6
	10c	V	For validation, describe how the predictions were calculated.	4-6
	10d	D;V	Specify all measures used to assess model performance and, if relevant, to compare multiple models.	6
	10e	V	Describe any model updating (e.g., recalibration) arising from the validation, if done.	n/a
Risk groups	11	D;V	Provide details on how risk groups were created, if done.	4–6
Development vs. validation	12	V	For validation, identify any differences from the development data in setting, eligibility criteria, outcome, and predictors.	4–6
			**Results**	
Participants	13a	D;V	Describe the flow of participants through the study, including the number of participants with and without the outcome and, if applicable, a summary of the follow-up time. A diagram may be helpful.	3
	13b	D;V	Describe the characteristics of the participants (basic demographics, clinical features, available predictors), including the number of participants with missing data for predictors and outcome.	6
	13c	V	For validation, show a comparison with the development data of the distribution of important variables (demographics, predictors and outcome).	6
Model development	14a	D	Specify the number of participants and outcome events in each analysis.	4
	14b	D	If done, report the unadjusted association between each candidate predictor and outcome.	4

Model specification	15a	D	Present the full prediction model to allow predictions for individuals (i.e., all regression coefficients, and model intercept or baseline survival at a given time point).	4
	15b	D	Explain how to the use the prediction model.	4
Model performance	16	D;V	Report performance measures (with CIs) for the prediction model.	8,9
Model-updating	17	V	If done, report the results from any model updating (i.e., model specification, model performance).	n/a
**Discussion**				
Limitations	18	D;V	Discuss any limitations of the study (such as nonrepresentative sample, few events per predictor, missing data).	11
Interpretation	19a	V	For validation, discuss the results with reference to performance in the development data, and any other validation data.	10
	19b	D;V	Give an overall interpretation of the results, considering objectives, limitations, results from similar studies, and other relevant evidence.	10
Implications	20	D;V	Discuss the potential clinical use of the model and implications for future research.	10
**Other information**				
Supplementary information	21	D;V	Provide information about the availability of supplementary resources, such as study protocol, Web calculator, and data sets.	n/a
Funding	22	D;V	Give the source of funding and the role of the funders for the present study.	n/a

* Items relevant only to the development of a prediction model are denoted by D, items relating solely to a validation of a prediction model are denoted by V, and items relating to both are denoted D;V. We recommend using the TRIPOD Checklist in conjunction with the TRIPOD Explanation and Elaboration document.

Table S2. STROBE Statement—Checklist of items that should be included in reports of cohort studies [2].

	Item No	Recommendation	Page(s)
**Title and abstract**	1	(*a*) Indicate the study's design with a commonly used term in the title or the abstract	1
		(*b*) Provide in the abstract an informative and balanced summary of what was done and what was found	1
**Introduction**			
Background/rationale	2	Explain the scientific background and rationale for the investigation being reported	1–2,
Objectives	3	State specific objectives, including any prespecified hypotheses	2
**Methods**			
Study design	4	Present key elements of study design early in the paper	2–4
Setting	5	Describe the setting, locations, and relevant dates, including periods of recruitment, exposure, follow-up, and data collection	2–4
Participants	6	(*a*) Give the eligibility criteria, and the sources and methods of selection of participants. Describe methods of follow-up	2-3
		(*b*) For matched studies, give matching criteria and number of exposed and unexposed	n/a
Variables	7	Clearly define all outcomes, exposures, predictors, potential confounders, and effect modifiers. Give diagnostic criteria, if applicable	4
Data sources/ measurement	8*	For each variable of interest, give sources of data and details of methods of assessment (measurement). Describe comparability of assessment methods if there is more than one group	3
Bias	9	Describe any efforts to address potential sources of bias	n/a
Study size	10	Explain how the study size was arrived at	2–3
Quantitative variables	11	Explain how quantitative variables were handled in the analyses. If applicable, describe which groupings were chosen and why	6
Statistical methods	12	(*a*) Describe all statistical methods, including those used to control for confounding	6
		(*b*) Describe any methods used to examine subgroups and interactions	n/a
		(*c*) Explain how missing data were addressed	3
		(*d*) If applicable, explain how loss to follow-up was addressed	n/a
		(*e*) Describe any sensitivity analyses	n/a
**Results**			

Participants	13*	(a) Report numbers of individuals at each stage of study—eg numbers potentially eligible, examined for eligibility, confirmed eligible, included in the study, completing follow-up, and analysed	3
		(b) Give reasons for non-participation at each stage	n/a
		(c) Consider use of a flow diagram	3
Descriptive data	14*	(a) Give characteristics of study participants (eg demographic, clinical, social) and information on exposures and potential confounders	7
		(b) Indicate number of participants with missing data for each variable of interest	n/a
		(c) Summarise follow-up time (eg, average and total amount)	7
Outcome data	15*	Report numbers of outcome events or summary measures over time	6
Main results	16	(*a*) Give unadjusted estimates and, if applicable, confounder-adjusted estimates and their precision (eg, 95% confidence interval). Make clear which confounders were adjusted for and why they were included	8–10
		(*b*) Report category boundaries when continuous variables were categorized	4
		(*c*) If relevant, consider translating estimates of relative risk into absolute risk for a meaningful time period	n/a
Other analyses	17	Report other analyses done—eg analyses of subgroups and interactions, and sensitivity analyses	n/a
**Discussion**			
Key results	18	Summarise key results with reference to study objectives	10
Limitations	19	Discuss limitations of the study, taking into account sources of potential bias or imprecision. Discuss both direction and magnitude of any potential bias	11
Interpretation	20	Give a cautious overall interpretation of results considering objectives, limitations, multiplicity of analyses, results from similar studies, and other relevant evidence	10–11
Generalisability	21	Discuss the generalisability (external validity) of the study results	12
**Other information**			
Funding	22	Give the source of funding and the role of the funders for the present study and, if applicable, for the original study on which the present article is based	n/a

Reference

1. Collins, G.S.; Reitsma, J.B.; Altman, D.G.; Moons, K.G.M. Transparent reporting of a multivariable prediction model for individual prognosis or diagnosis (TRIPOD) the TRIPOD statement. *Circulation* **2015**, *131*, 211–219.
2. Vandenbroucke, J.P.; Von Elm, E.; Altman, D.G.; Gøtzsche, P.C.; Mulrow, C.D.; Pocock, S.J. The strengthening the reporting of observational studies in epidemiology (strobe) statement: Guidelines for reporting. *Ann. Intern. Med.* **2007**, *147*, 573–578.
